# Ask, and it shall be given you – individual patient data and code availability for randomised controlled trials submitted for publication

**DOI:** 10.1111/anae.16503

**Published:** 2024-12-05

**Authors:** Paul Bramley

**Affiliations:** ^1^ Department of Anaesthesia Sheffield Teaching Hospitals NHS Foundation Trust Sheffield UK

Sharing data from clinical studies is now recognised to be an important part of the research process [1]. Since many research results cannot be replicated [2, 3], there has been growing interest in making study documents available [4, 5] in order to make reproduction of existing results, detection of false results, and replication of findings and synthesis into larger meta‐studies easier. Randomised controlled trials (RCTs) are of particular interest, since they are expensive and time‐consuming to run. Post‐publication availability of study documents has been investigated previously, but their availability to journals at the point of manuscript submission, where it could be used as part of the review, has not been evaluated.

To address this, for a 9‐month period (1 June 2023 to 29 February 2024), when an RCT was submitted to *Anaesthesia* and sent for peer review (i.e. not desk rejected), a member of the editorial team requested, via email, anonymised individual patient data (IPD) and statistical code from the corresponding author. We sent one further request if there was no initial response. I examined the submitted manuscript and any provided IPD and code for each RCT to determine: whether the IPD and code were stated to be available in the submitted manuscript; whether the IPD and code were provided on request to the authors by the journal; the IPD format (if it was provided in multiple formats, the least proprietary format was recorded); whether there was a data dictionary; whether IPD were presented in English; whether (using the manuscript and/or Google Translate) it was clear what the variable names in the IPD represented; whether the results of the manuscript could theoretically be reproduced with the provided documents (I did not actually compare the values); and whether authors changed their submitted manuscript based on the request for IPD. I judged reproducibility was ‘possible’ if code and IPD were available, unless a fully reproducible document was available (e.g. R Markdown). For the proprietary files that could contain code but I was unable to open, I labelled code availability ‘unclear’. The project was approved by the editorial board of *Anaesthesia*, and the host institution confirmed that ethical approval was not required given that IPD were anonymised by authors before transfer. I performed all data cleaning and analysis in R (R Foundation, Vienna, Austria) and all analysis was exploratory.

**Figure 1:**
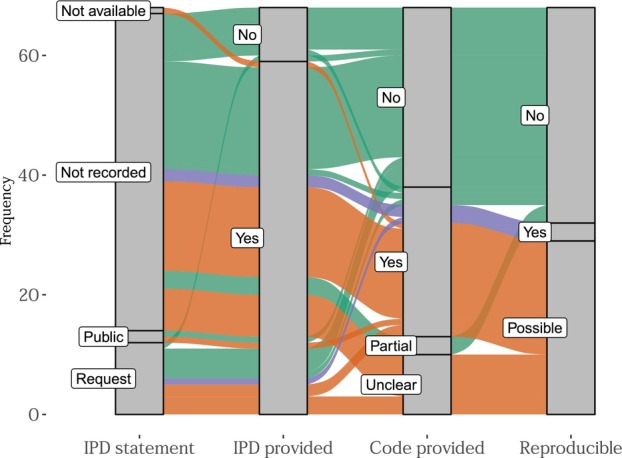
Alluvial plot showing features of manuscripts. Based on the submitted documents blue studies were reproducible, orange studies were potentially reproducible, and green studies were not reproducible. IPD, individual patient data.

In the 9‐month data collection window 122 RCTs were submitted to *Anaesthesia*, 44 of which were desk rejected. Of the remaining 78, we missed the opportunity to request IPD for eight before the manuscripts were rejected. Two provided IPD in such a way that we could not access them (one because of concerns about malware, another due to access issues with a website) and so were also excluded. This left a cohort of 68 manuscripts for further analysis. After we requested data (without any other prompting), authors of six (9%) RCTs reported finding errors in their manuscript which they wished to correct. Nine RCTs provided no IPD (see Table 1 and Fig. 1), of which one refused citing ethical concerns and one refused to provide it unless the manuscript was accepted. One authorship group withdrew their submission after the data request (without providing data).

**Table 1:** Summary statistics for collected variables.

	n = 68
IPD availability stated in manuscript	
Not recorded	53 (78%)
On request	12 (18%)
Public database	2 (3%)
Stated not to be available	1 (1%)
Code availability stated in manuscript	
Not recorded	66 (97%)
Public database	1 (1%)
On request	1 (1%)
IPD provided	59 (87%)
Code provided	
No	31 (46%)
Yes	24 (35%)
Unclear	10 (15%)
Partial	3 (4%)

	n = 59
IPD format	
CSV	3 (5%)
Excel	51 (86%)
SPSS	3 (5%)
Stata	2 (3%)
Data dictionary	
No	42 (71%)
Partial	12 (20%)
Yes	5 (8%)
IPD in English	
Yes	47 (80%)
Partial	5 (8%)
No	7 (12%)
IPD variables clearly labelled	54 (92%)
Reproducible	
No	27 (46%)
Possible	29 (49%)
Yes	3 (5%)

IPD, individual patient data.

In contrast to previous studies investigating whether IPD were provided on request [6], I found that despite most manuscripts not making a statement about data availability, 87% of authors would provide IPD on request. However, this could be explained by the incentives for authors to provide IPD to a journal as part of a review, rather than to other researchers post‐publication. This is relevant since previous work on trial submissions has shown that many problems with data integrity required IPD to be detected [7]. Fewer authors provided statistical code, which was a surprise given that this has fewer confidentiality implications and is straightforward in most statistical packages. This may be due to lack of technical expertise, which is supported by the fact that several groups provided documents labelled as code which were not statistical code. Despite this, more than half of submissions provided documents which could lead to full reproducibility of results – though I lacked the resources to evaluate whether all results were reproducible. It is also notable that 9% of all submissions found errors in their own work following a request for IPD. This suggests that the expectation of scrutiny causes authors to check their work and makes routine requests for IPD seem a potentially valuable tool for journals. Finally, it was reassuring that most datasets could be opened with freely available tools and variable names could be interpreted in context, despite the limited availability of data dictionaries.
